# Supplementary material for: Flow Synthesis of L-α-Glycerylphosphorylcholine: Studies on Synthetic Routes Applicable to a Flow Reactor and Optimization of Reaction Conditions
Source: Pharmaceutics. 2022 Nov 16;14(11):2480. doi: 10.3390/pharmaceutics14112480 (PMC9692846; doi:10.3390/pharmaceutics14112480)
Supplement: Supplementary file 1 [file pharmaceutics-14-02480-s001.zip › pharmaceutics-1972399-supplementary.pdf]

Supporting Information for

Flow Synthesis of L-α-Glycerylphosphorylcholine: Studies on Synthetic Routes Applicable to a Flow Reactor and Optimization of Reaction Conditions

Jihun Park [1], Seungjae Lee [2], Gyungtak Kim [1], Yashwardhan R. Malpani [2], Boyoung Y. Park [3] and Ye-Jin Hwang [1,*]

1 Department of Chemistry and Chemical Engineering, Education and Research Center for Smart Energy and Materials, Inha University, 100 Inha-ro, Michugol-gu, Incheon 22212, Republic of Korea
2 API Synthesis Team, Daewoong-Bio Incorporated, 29-Jeyakdanji-ro, Hyangnam-eup, Hwaseong-si 18608, Republic of Korea
3 College of Pharmacy, Kyung Hee University, 26 Kyughee-daero, Dongdaemun-gu, Seoul 02447, Republic of Korea

[*] *Correspondence: yjhwang@inha.ac.kr; Tel.: +82-32-860-7464*

Material synthesis

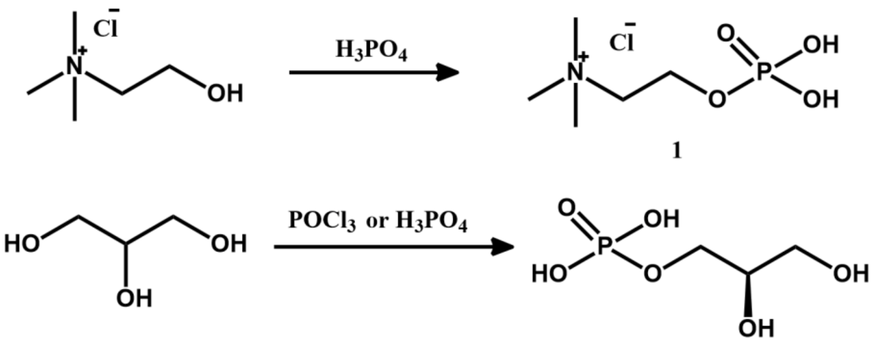

Scheme S1. First step reactions to prepare L-α-GPC.

N,N,N-trimethyl-2-(phosphonooxy)ethanaminium chloride (compound 1)

Choline chloride (50 g, 0.358 mol) and phosphoric acid (100 g, 1.02 mol) were mixed in a flask equipped with a Dean-Stark trap. The solution was mixed during warming up to 170 °C for 6 hours. After the reaction was ended, the reaction mixture was dried in vacuo and phosphocholine (0.286 mol, 80%) was obtained as colorless liquid.

(R)-2,3-dihydroxypropyl dihydrogen phosphate (compound 2)

Glycerol (32.8 g, 0.358 mol) and phosphoric acid (100 g, 1.02 mol) were mixed in a flask equipped with a Dean-Stark trap. The solution was mixed during warming up to 170 °C for 6 hours. After the reaction was ended, the reaction mixture was dried in vacuo but (R)-2,3-dihydroxypropyl dihydrogen phosphate was not synthesized.

(R)-2,3-dihydroxypropyl dihydrogen phosphate (compound 2)

$POCl_3$ (1.75 g, 12.5 mmol) and 2 mL of anhydrous chloroform were mixed in a flask under nitrogen atmosphere and stirred for 10 min at room temperature. Glycerol (0.132 mg, 1.44 mmol) was slowly added to the flask and the reaction was carried out at room temperature for 15 hours. After the reaction was ended, the reaction mixture was quenched by adding deionized water to remove unreacted $POCl_3$. The solution was dried in vacuo at 60 °C, but (R)-2,3-dihydroxypropyl dihydrogen phosphate was not synthesized.

NMR data

Table S1. Optimized reaction conditions and conversions of first step reactions in a batch reactor.

Entry	reactant	phosphorylation reagent	solvent	temperature (°C)	reaction time (hr)	conversion [a] (%)
1-1	choline chloride	$POCl_3$	$H_2O/CHCl_3$	25	4	86
1-2	Choline chloride	H_3PO_4	-	170	6	< 20
1-3	choline chloride	H_3PO_4	-	170	6	80
1-4	glycerol	$POCl_3$	$CHCl_3$	25	15	-
1-5	glycerol	H_3PO_4	-	170	6	-

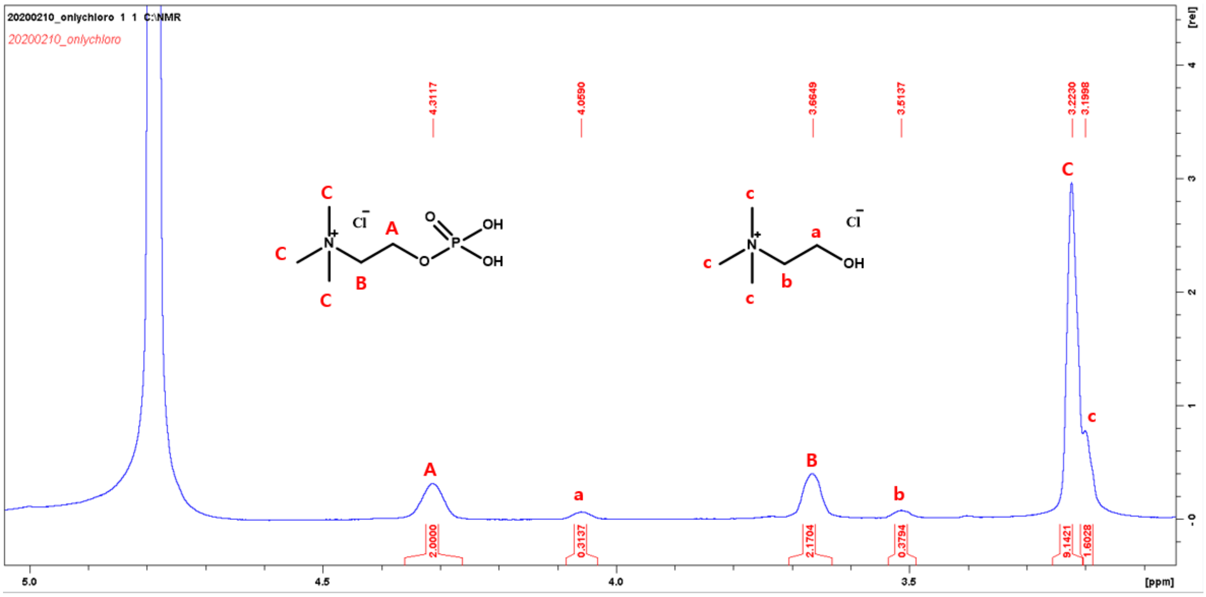

Figure S1. ^{1}H NMR spectrum of **entry 1-1.**

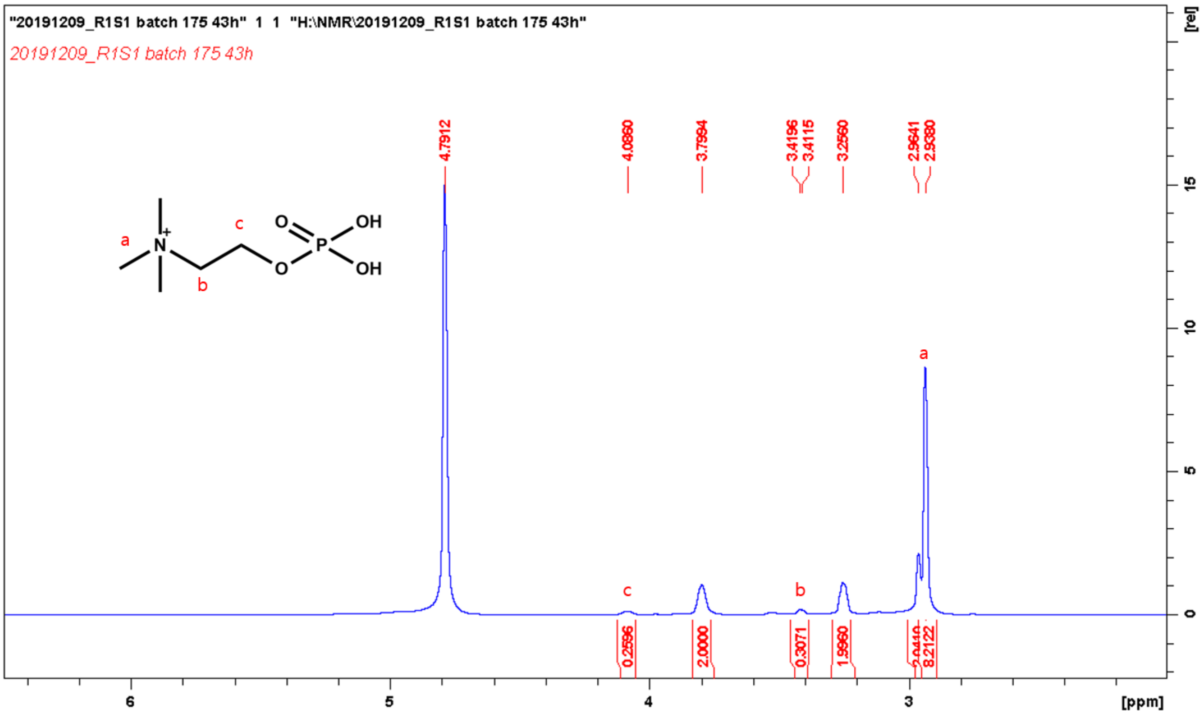

Figure S2. ^{1}H NMR spectrum of **entry 1-2.**

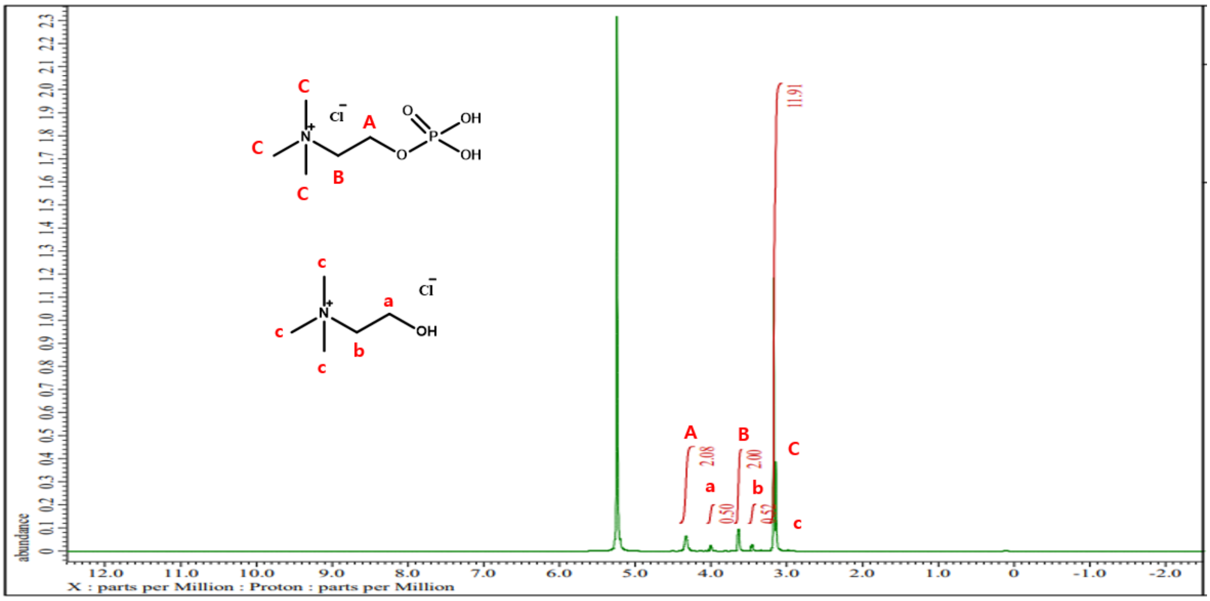

Figure S3. ^{1}H NMR spectrum of **entry 1-3.**

Table S2. Reaction conditions and conversions of first step reactions in a flow reactor.

entry	H_2O [a] (mL)	$POCl_3$/ choline chloride [b]	reactor volume (mL)	temperature (°C)	flow rate (μL/min)	reaction time	Conversion [c] (%)
2-1	0.3	8	22.5	25	31.25	1hr 38min	60
2-2	0.3	8	22.5	0	31.25	4hr	8
2-3	0.3	8	22.5	30	31.25	35min	32
2-4	0.3	8	22.5	35	31.25	30min	38
2-5	0.3	6	22.5	25	31.25	1hr 56min	88
2-6	0.3	4	22.5	25	31.25	2hr 24min	36
2-7	0.15	6	30	25	31.25	3hr 25min	97

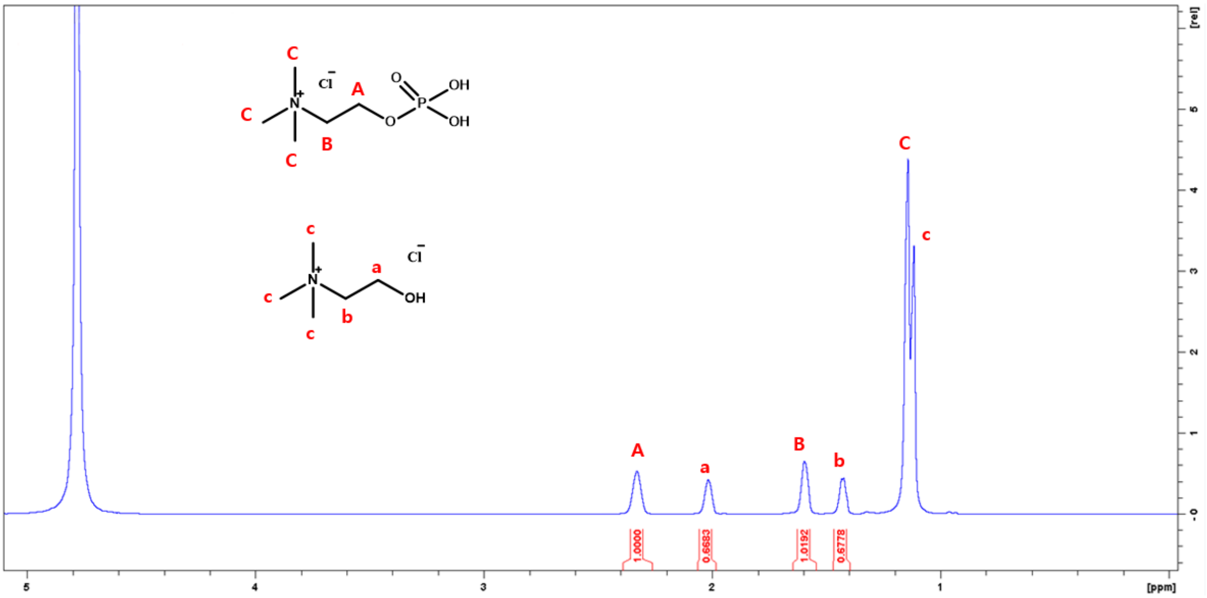

Figure S4. ^{1}H NMR spectrum of entry **2-1.**

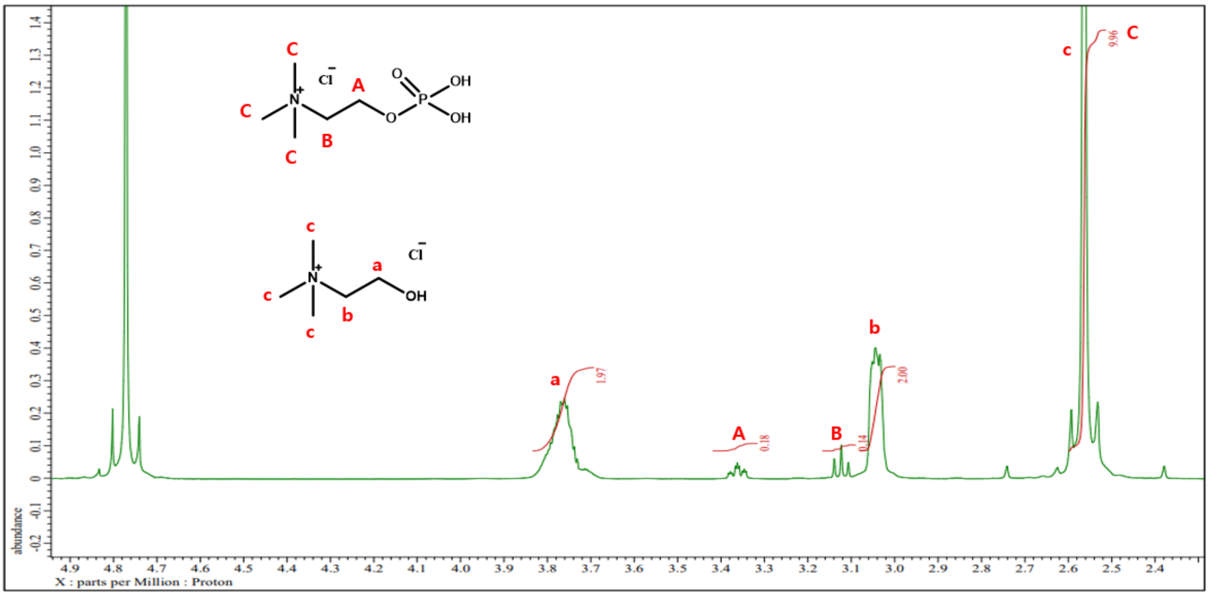

Figure S5. ^{1}H NMR spectrum of entry **2-2.**

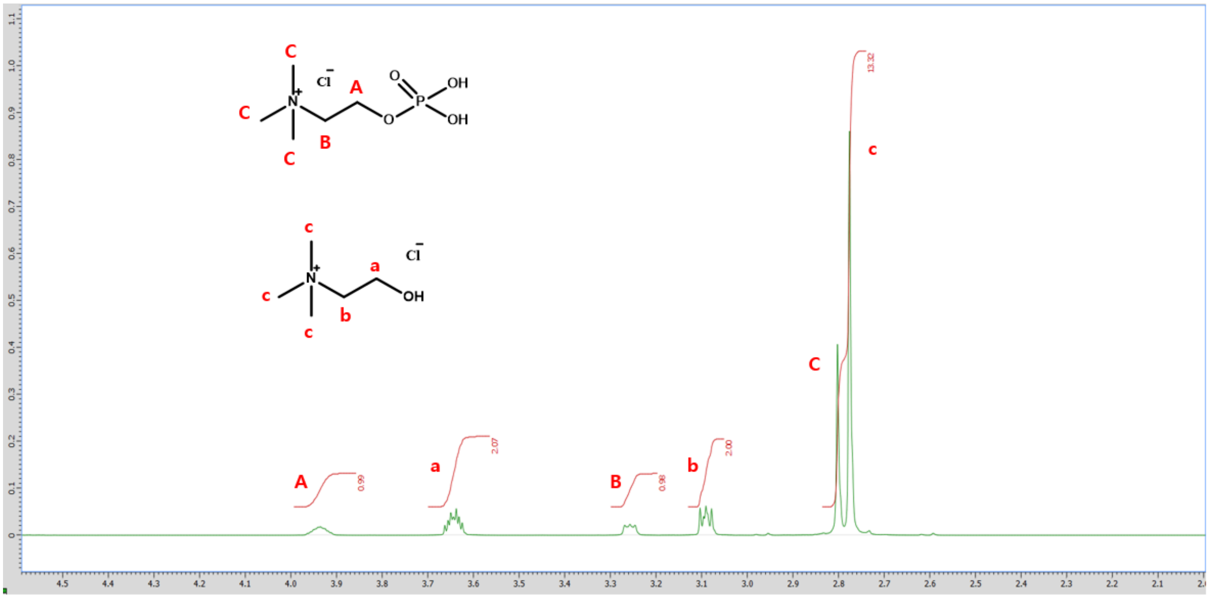

Figure S6. ^{1}H NMR spectrum of **entry 2-3.**

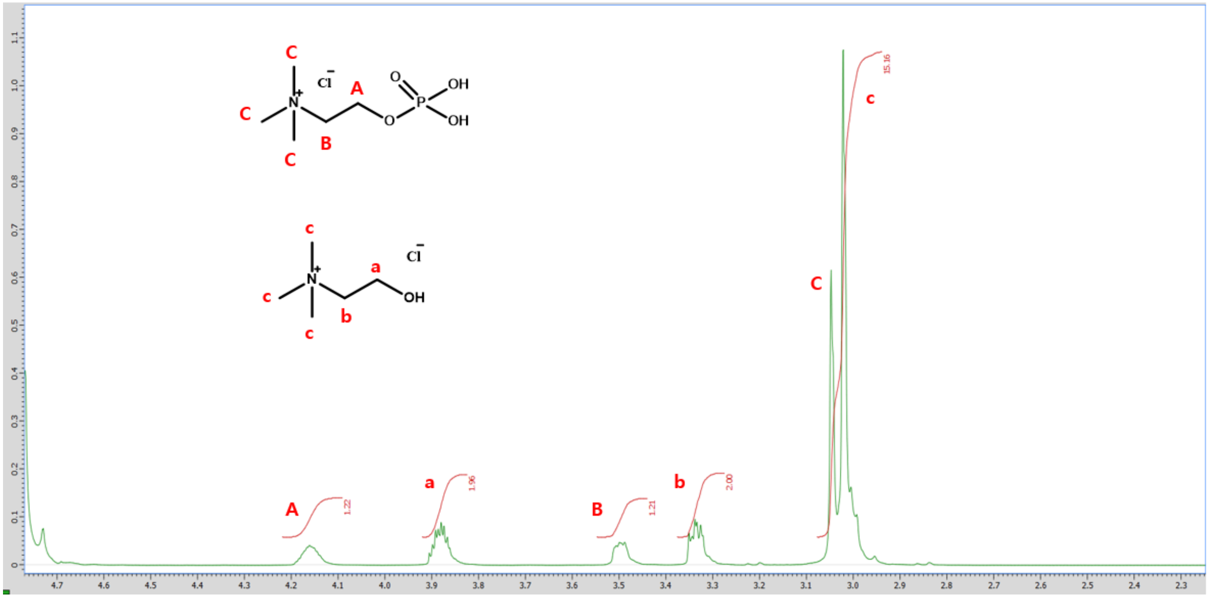

Figure S7. ^{1}H NMR spectrum of entry **2-4.**

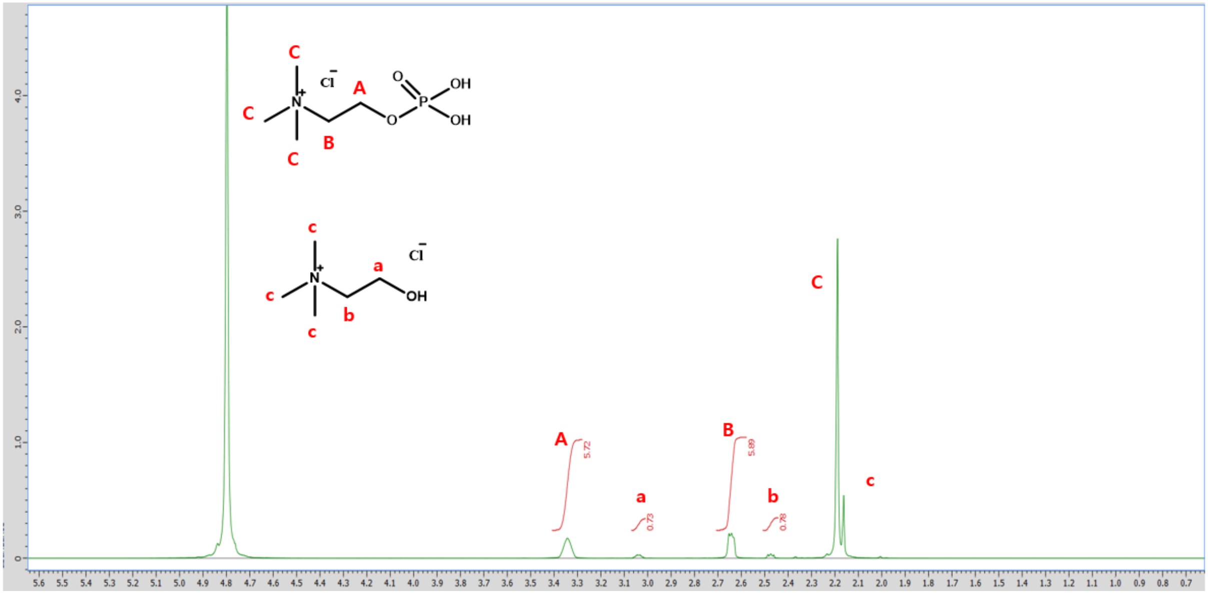

Figure S8. ^{1}H NMR spectrum of entry **2-5.**

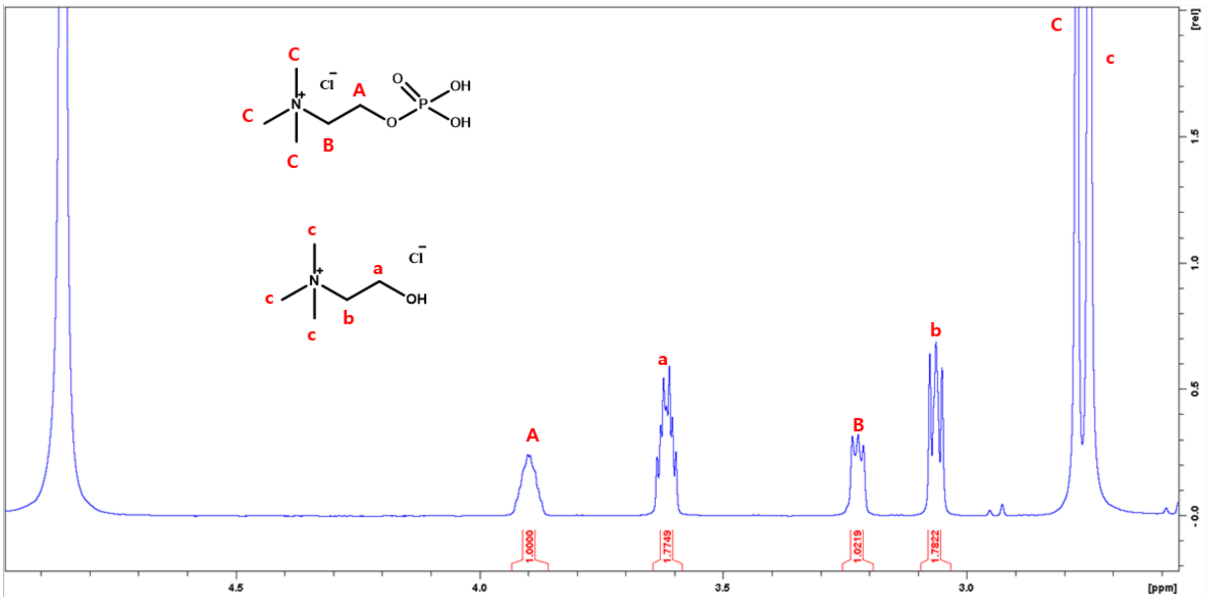

Figure S9. ^{1}H NMR spectrum of **entry 2-6.**

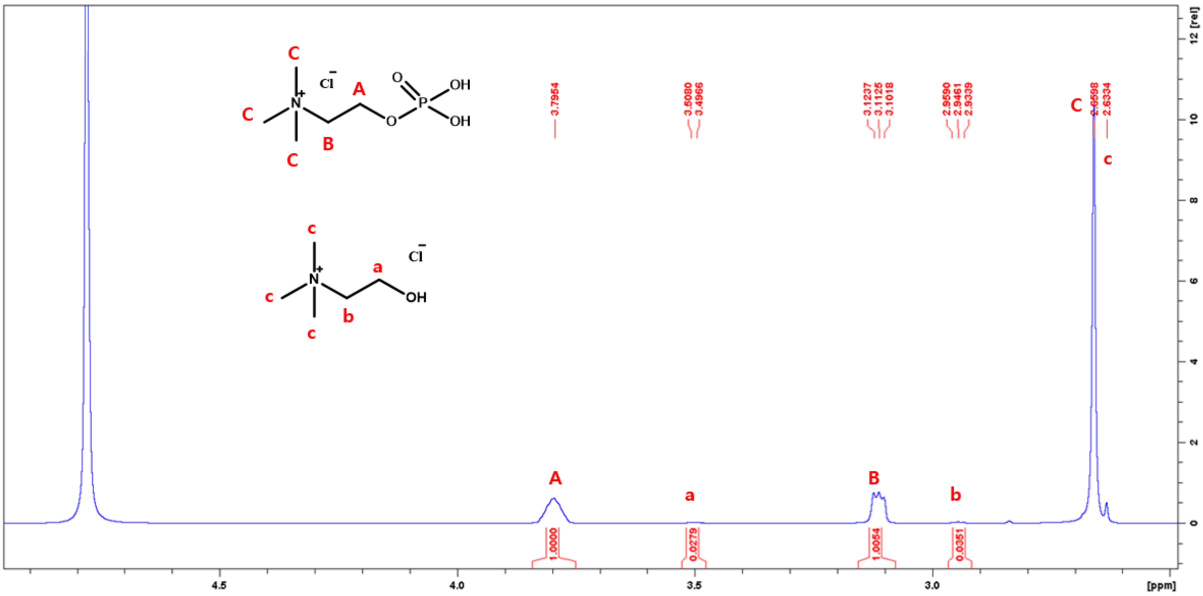

Figure S10. ^{1}H NMR spectrum of entry **2-7.**

Table S3. Reaction conditions and conversions of second step reactions in a batch reactor.

entry	solvent	CPD/ phosphorylcholine	temperature (°C)	reaction time	conversion (%)
3-1	ethanol	1.14	75	24hr	90
3-2	H_2O + ethanol	1.14	75	24hr	98

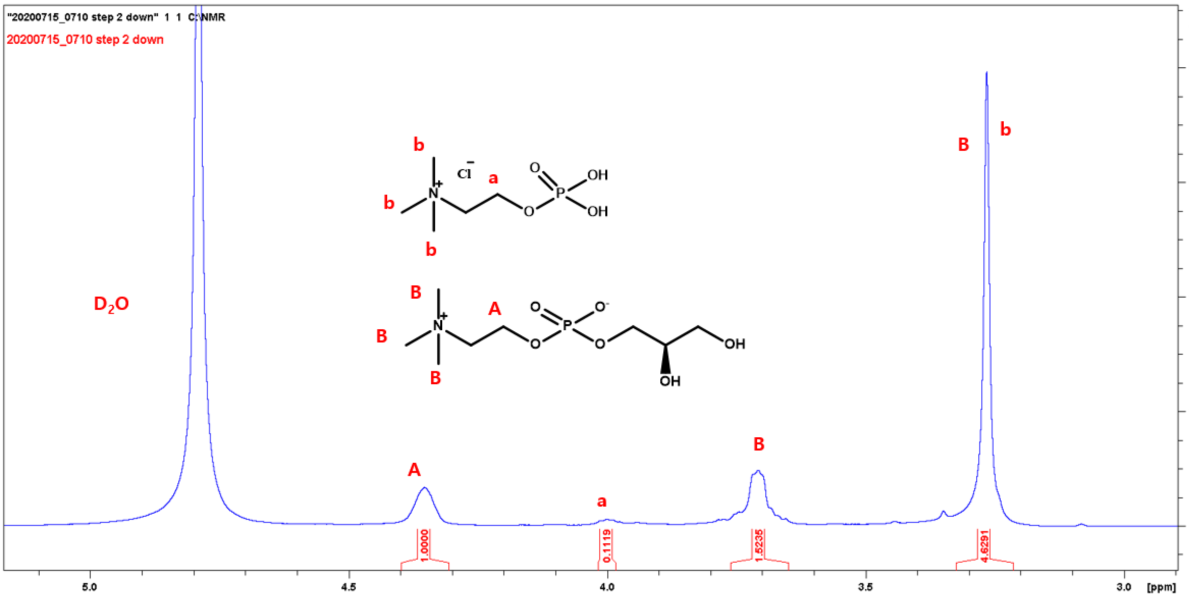

Figure S11. ¹H NMR spectrum of entry **3-1.**

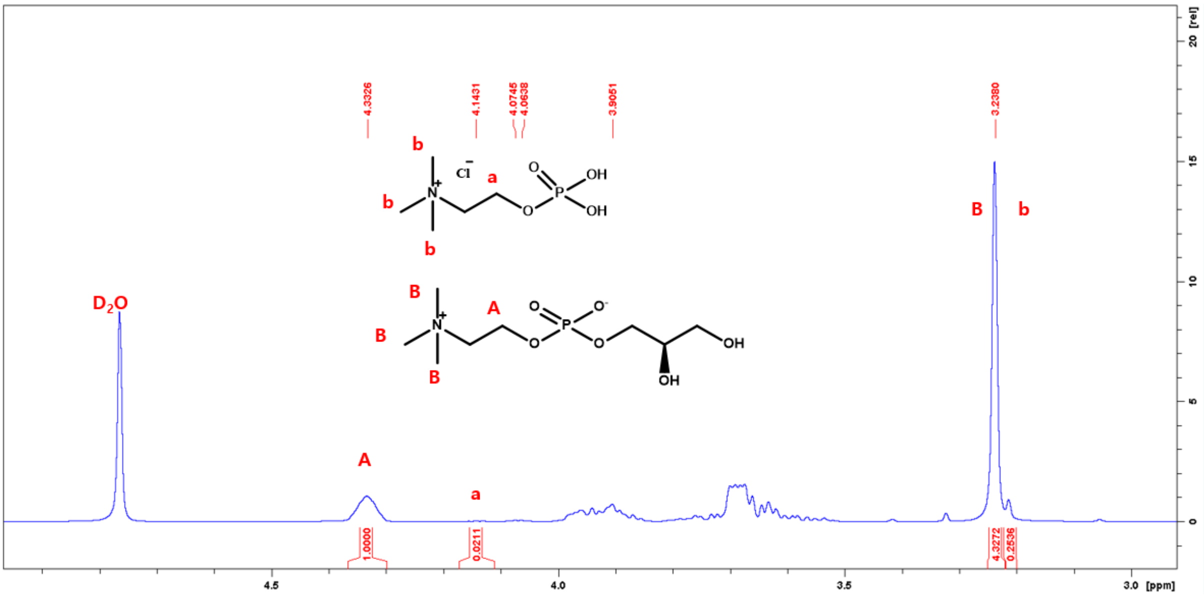

Figure S12. ¹H NMR spectrum of entry **3-2.**

Table S4. Reaction conditions and conversions of second step reactions in a flow reactor.

entry	H_2O (mL)	reactor volume (mL)	temperature (°C)	reaction time	Conversion (%)
4-1	15	12	75	6hr	30
4-2	12	12	75	6hr	42
4-3	10	12	75	6hr	61
4-4	10	12	80	6hr	79

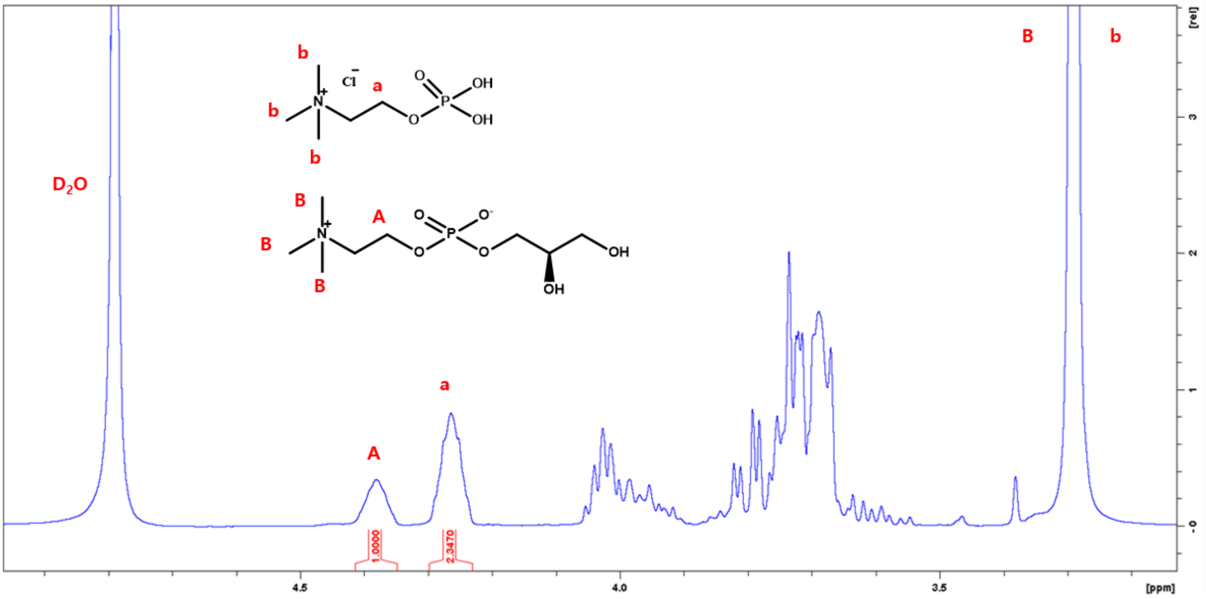

Figure S13. ^{1}H NMR spectrum of entry **4-1.**

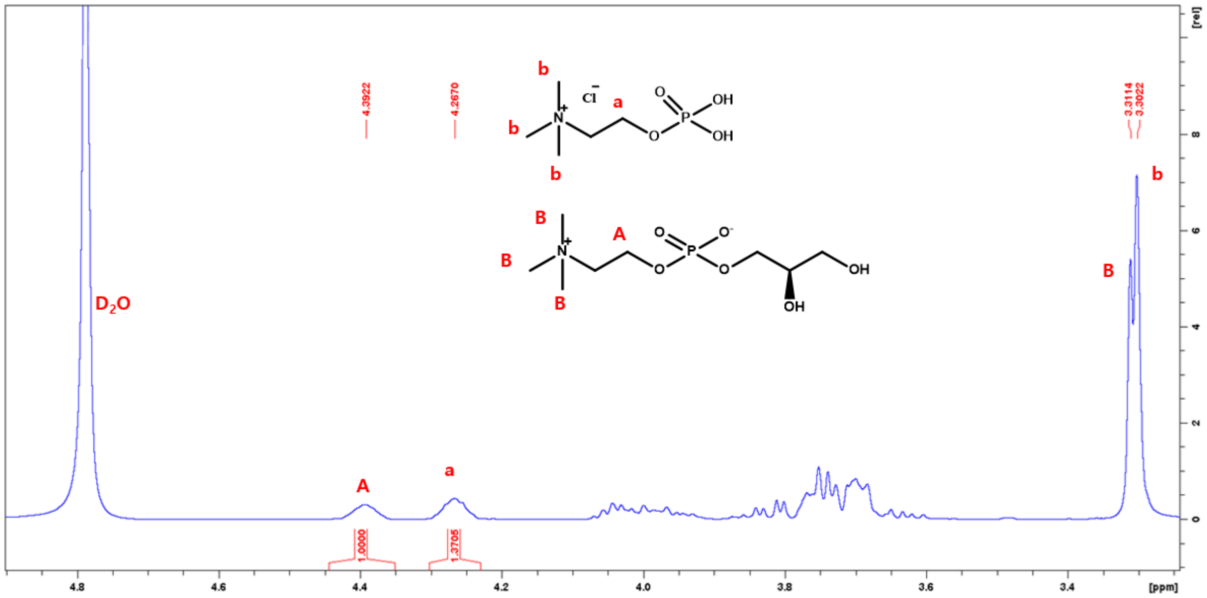

Figure S14. ^{1}H NMR spectrum of entry **4-2.**

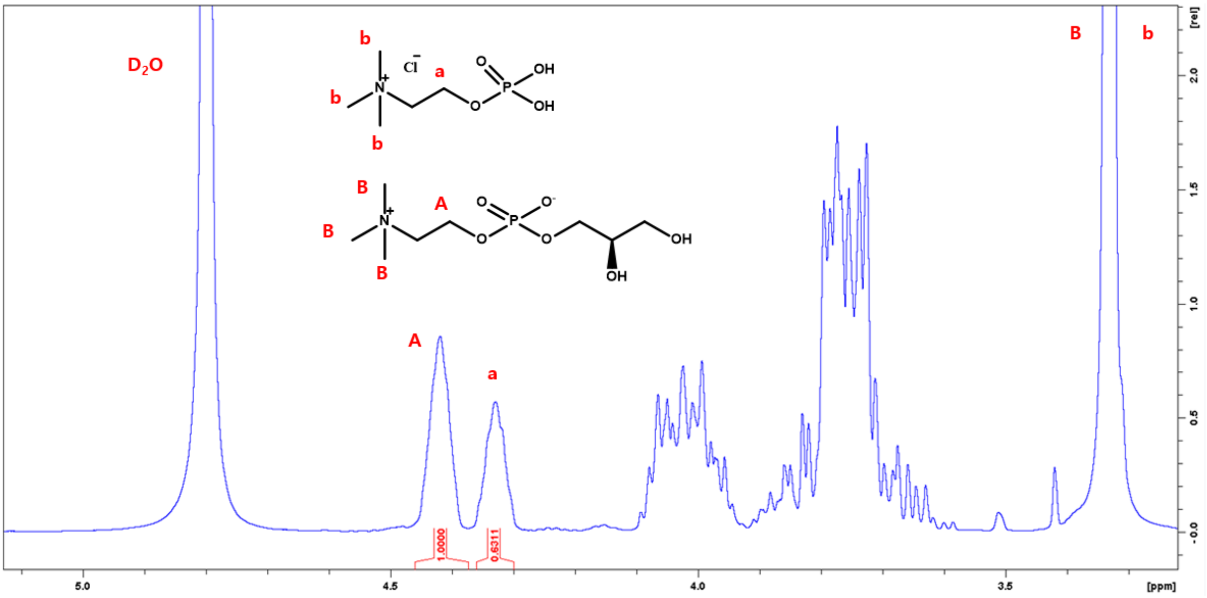

Figure S15. ^{1}H NMR spectrum of entry **4-3.**

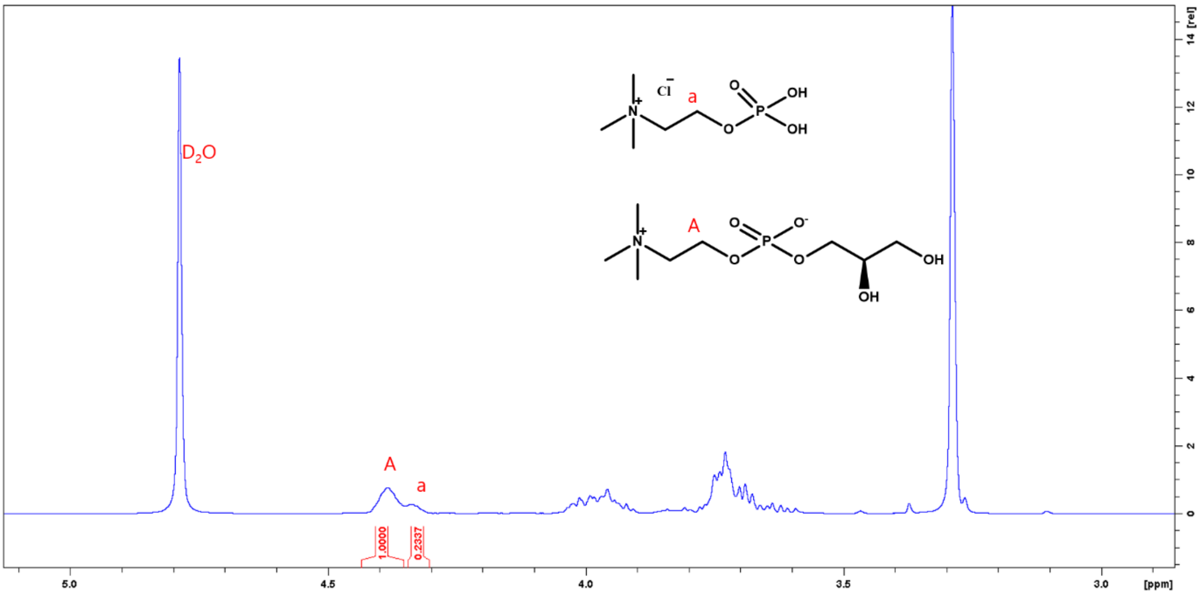

Figure S16. ^{1}H NMR spectrum of entry **4-4.**

Table S5. Reproducibility experiment of first step reactions in a flow reactor.

entry	H_2O [a] (mL)	$POCl_3$/ choline chloride [b]	reactor volume (mL)	temperature (°C)	flow rate (μL/min)	reactn time	Conversion [c] (%)
1	0.15	6	30	25	31.25	3hr 25min	97
2	0.15	6	30	25	31.25	3hr 13min	95.2
3	0.15	6	30	25	31.25	3hr 26min	97.3

Table S6. Reproducibility experiment of second step reactions in a flow reactor.

entry	H_2O (mL)	reactor volume (mL)	temperature (°C)	reaction time	Conversion (%)
1	10	12	80	6hr	79
2	10	12	80	6hr	76
3	10	12	80	6hr	78

^{13}C NMR

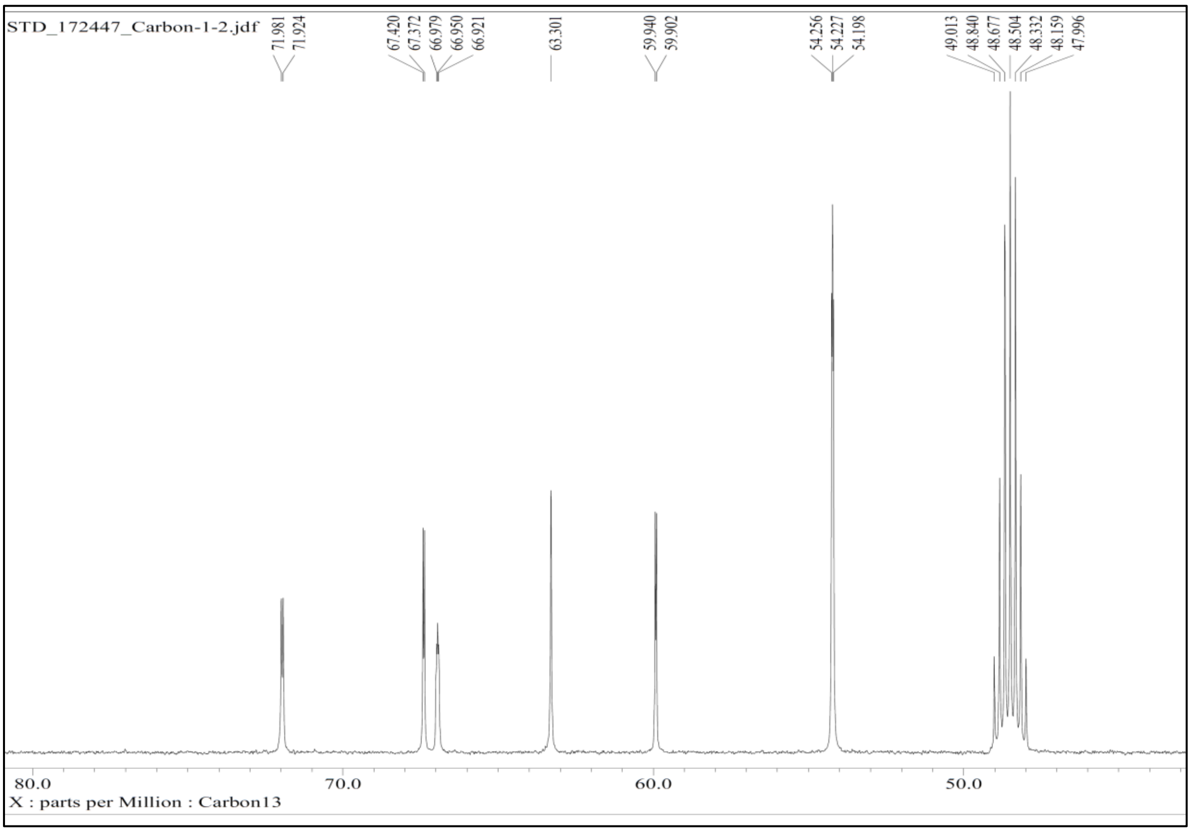

Figure S17. ^{13}C NMR spectrum of choline alfoscerate.

Mass spectrum

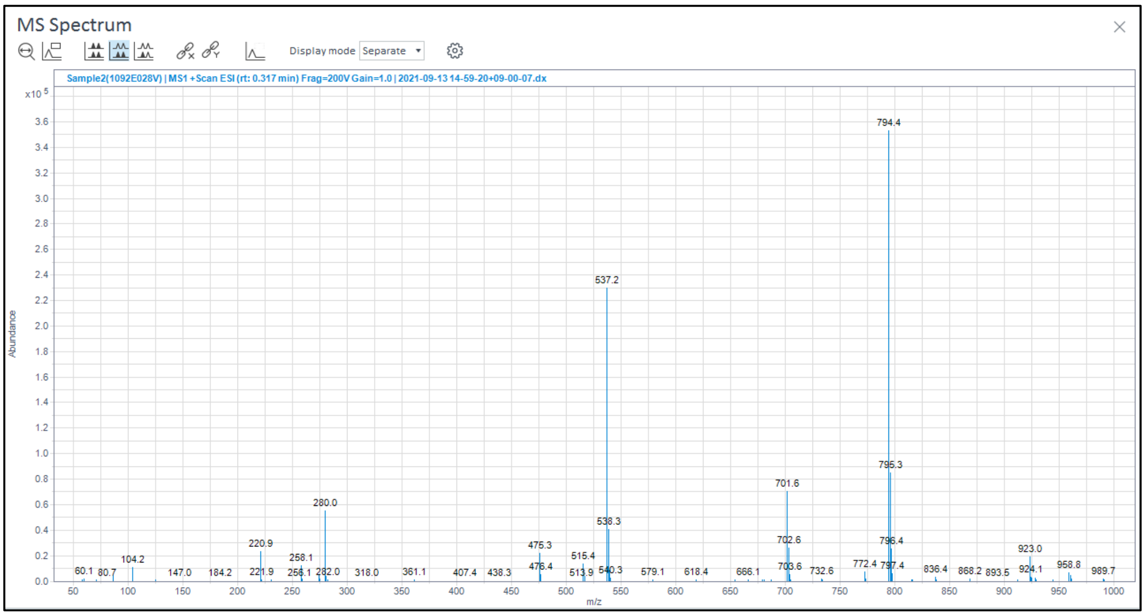

Figure S18. Mass spectrum of choline alfoscerate.

IR spectrum

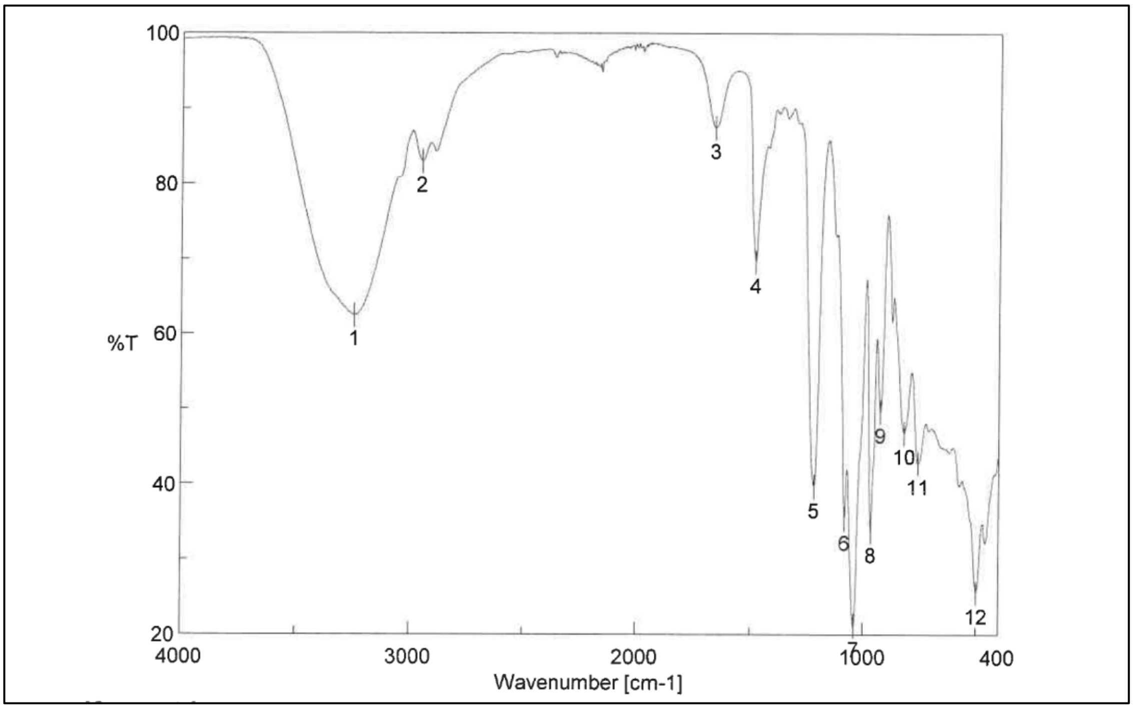

Figure S19. IR spectrum of choline alfoscerate.

HPLC data

Data file : D:\Chem32\1\Data\c-CAC\c-CAC 2021-11-16 14-28-28\020-16- ->
Sample Name: 1092B032 1

Seq Line : 20 Inj. No. : 1
Location : 16 Inj. Vol. : 100 µl
Injection Date : 11/17/2021 10:05:08 AM
Acq. Method : D:\Chem32\1\Data\c-CAC\c-CAC 2021-11-16 14-28-28\c- ->
Analysis Method : D:\Chem32\1\Data\c-CAC\c-CAC 2021-11-16 14-28-28\c- ->
Last Changed : 12/6/2021 10:54:06 AM

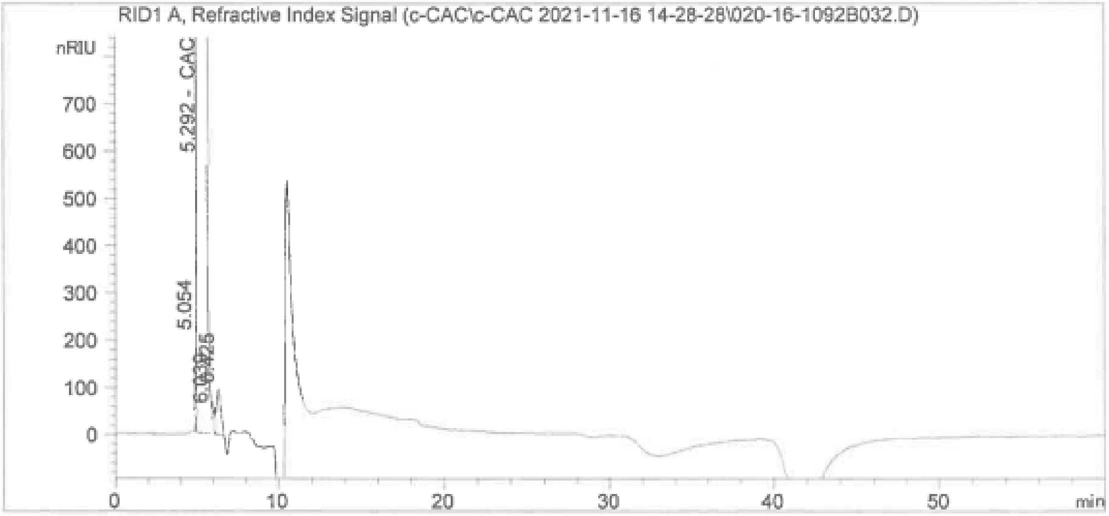

Customized Report: DATA PRINT

Sorted By Signal
Calib. Data Modified : 12/6/2021 10:53:39 AM
Multiplier : 1.000000
Dilution : 1.000000
Uncalibrated Peaks : not reported
Signal 1: RID1 A, Refractive Index Signal

Peak #	RT [min]	Type	Width [min]	Area	Area %	Name
1	5.054	MF	0.072	879.973	0.062	
2	5.292	FM	0.251	1421880.875	99.777	CAC
3	6.039	FM	0.166	525.062	0.037	
4	6.425	FM	0.307	1775.576	0.125	

*** End of Report ***

Figure S20. HPLC graph of choline alfoscerate.
